# Computer-Aided Targeting of the PI3K/Akt/mTOR Pathway: Toxicity Reduction and Therapeutic Opportunities

**DOI:** 10.3390/ijms151018856

**Published:** 2014-10-20

**Authors:** Tan Li, Guanyu Wang

**Affiliations:** Department of Biology, South University of Science and Technology of China, 1088 Xueyuan Rd., Shenzhen 518055, China; E-Mail: li.t@sustc.edu.cn

**Keywords:** PI3K/Akt/mTOR pathway, computer-aided targeting, mathematical modeling, molecular dynamics simulation, molecular targeted therapy, combination therapy, metabolic disorder

## Abstract

The PI3K/Akt/mTOR pathway plays an essential role in a wide range of biological functions, including metabolism, macromolecular synthesis, cell growth, proliferation and survival. Its versatility, however, makes it a conspicuous target of many pathogens; and the consequential deregulations of this pathway often lead to complications, such as tumorigenesis, type 2 diabetes and cardiovascular diseases. Molecular targeted therapy, aimed at modulating the deregulated pathway, holds great promise for controlling these diseases, though side effects may be inevitable, given the ubiquity of the pathway in cell functions. Here, we review a variety of factors found to modulate the PI3K/Akt/mTOR pathway, including gene mutations, certain metabolites, inflammatory factors, chemical toxicants, drugs found to rectify the pathway, as well as viruses that hijack the pathway for their own synthetic purposes. Furthermore, this evidence of PI3K/Akt/mTOR pathway alteration and related pathogenesis has inspired the exploration of computer-aided targeting of this pathway to optimize therapeutic strategies. Herein, we discuss several possible options, using computer-aided targeting, to reduce the toxicity of molecularly-targeted therapy, including mathematical modeling, to reveal system-level control mechanisms and to confer a low-dosage combination therapy, the potential of PP2A as a therapeutic target, the formulation of parameters to identify patients who would most benefit from specific targeted therapies and molecular dynamics simulations and docking studies to discover drugs that are isoform specific or mutation selective so as to avoid undesired broad inhibitions. We hope this review will stimulate novel ideas for pharmaceutical discovery and deepen our understanding of curability and toxicity by targeting the PI3K/Akt/mTOR pathway.

## 1. The PI3K/Akt/mTOR Pathway

**Figure 1:**
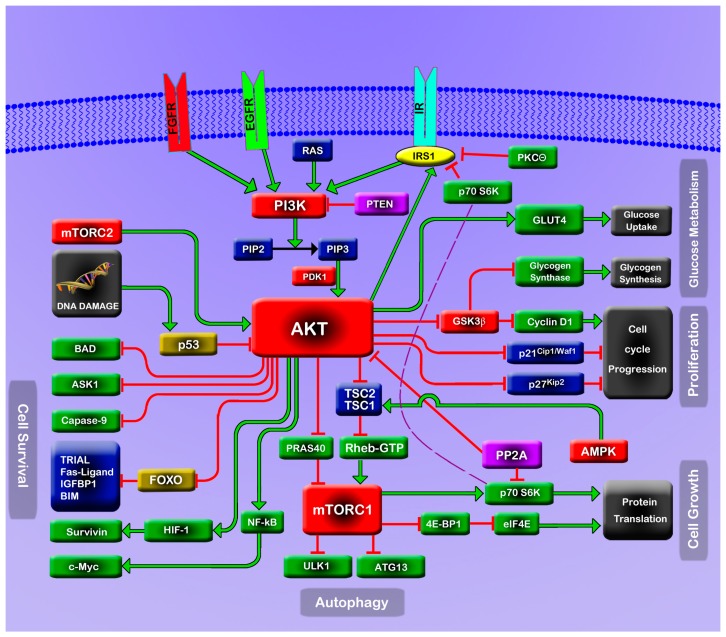
A schematic representation of the PI3K/Akt/mTOR pathway.

The PI3K/Akt/mTOR pathway (Figure 1) is a major cellular signaling pivot in the cellular response to extracellular stimuli, including insulin, insulin-like growth factor-1 (IGF-1), epidermal growth factor (EGF) and fibroblast growth factor (FGF). For example, upon insulin stimulation, the insulin receptor phosphorylates insulin receptor substrate 1 (IRS1) at tyrosine sites, which, in turn, activates phosphatidylinositol-3 kinase (PI3K). PI3K then converts phosphatidylinositol-4,5-bisphosphate (PIP2) into phosphatidylinositol-3,4,5-trisphosphate (PIP3). PIP3 recruits Akt (also known as PKB; namely protein kinase B) to the membrane, where Akt is phosphorylated by the enzyme PDK1 at Thr^308^ and by rictor-bound mTOR complex 2 (mTORC2) at Ser^473^, for full Akt activation. Conversely, the phosphorylated Akt (pAkt) can be dephosphorylated by protein phosphatase 2A (PP2A) and the pleckstrin homology domain leucine-rich repeat protein phosphatase (PHLPP) [1]. Although PP2A may preferentially dephosphorylate Akt on Thr^308^, it can also dephosphorylate Akt on Ser^473^ under certain conditions [2]. PHLPP specifically dephosphorylates Akt on Ser^473^ [2]. The activated Akt further activates raptor-bound mTOR complex 1 (mTORC1) by two mechanisms. One is through inhibitory phosphorylation of tuberous sclerosis complex 2 (TSC2). Inhibited TSC2 blockades the complex of TSC1/TSC2 transforming Rheb-GTP to Rheb-GDP, resulting in the activation of mTORC1 by Rheb-GTP. The other is through Akt inhibition of proline-rich AKT substrate 40 (PRAS40), which inhibits mTORC1 [1,3]. The activated mTORC1, in turn, mediates phosphorylation of 4E-BP1 and p70S6K (S6K). Of note, S6K phosphorylates Ser^312^ and Ser^636/639^ at the *C* terminus of IRS1, which weakens insulin-stimulated tyrosine phosphorylation of IRS1 at this region and, thus, weakens its ability to bind PI3K. This event thus completes a negative feedback from Akt to IRS1 [4]. On the other hand, Akt directly phosphorylates IRS1 at Ser^629^ to strengthen its activity, thus creating a positive feedback from Akt to IRS1 [5].

The PI3K/Akt/mTOR pathway carries out a large spectrum of cellular functions [3,6]. For instance, mTORC1 acts as a major sensor of nutrient levels, energy levels and stress signals. mTORC1 promotes protein synthesis and cell growth by phosphorylating its immediate downstream targets, S6K and 4E-BP1, which regulate mRNA translation initiation and progression [6]. It also phosphorylates ATG13 and ULK1, to blockade the initiation of autophagy [6]. When the energy level is low, inhibitory signals from 5' AMP-activated protein kinase (AMPK) target mTORC1 to slow down translation. Similarly, when the cell is under stress, such as hypoxia and DNA damage, signals from REDD1 and p53 act to inhibit mTORC1 [6]. Akt is the essential signaling hub for the growth and survival of the cell, due to the broad spectrum of critical cellular functions it commands. Aside from dominantly activating mTORC1, Akt conveys pro-survival signals by phosphorylating and inactivating pro-apoptotic proteins, such as BAD and ASK1; it also downregulates the transcription of several pro-apoptotic genes, including tumor necrosis factor-related apoptosis-inducing ligand (TRAIL), Fas ligand, immunoglobulin-binding protein-1 (IGFBP1) and Bim, through inhibitory phosphorylation of transcription factors, including the Forkhead box O (FoxO) family [7]. Not only can Akt counteract apoptosis by downregulating pro-apoptotic signals, it can also directly phosphorylate and inhibit caspase-9, which is essential for caspase-dependent apoptosis [3]. Activated Akt also promotes cell survival through coupling with the NF-κB pathway. However, excessive stimulation of this pathway branch can lead to the expression of proto-oncogene c-Myc, contributing to oncogenesis and resistance to chemotherapy [8,9]. Akt also promotes cell cycle progression by promoting cyclin D1 expression and inhibitory phosphorylation of the cyclin-dependent kinase inhibitors, p21^Cip1/Waf1^ and p27^Kip2^ [7,10]. In the regulation of plasma glucose concentration, the process of Akt recruiting glucose transporter 4 (GLUT4) to the cell membrane is one of the most important events in the response of fat and muscle cells to insulin; active GLUT4 in the cell membrane is responsible for high-efficiency glucose uptake, and aberration of this mechanism is a critical component of insulin-resistance, the underlying pathology of type 2 diabetes (T2D) [6]. Moreover, Akt phosphorylates and inactivates glycogen synthase kinases 3 (GSK3α and GSK3β), shifting cells from catabolic to anabolic states [3].

Since the PI3K/Akt/mTOR pathway plays such a prominent role in the signaling network, it must be subject to strict internal regulations (e.g., by the feedback loops, as already introduced) and external regulations (e.g., by crosstalk with other signaling pathways, such as the Ras/MEK/ERK- and AMPK-related pathway [11]). PP2A, a tumor suppressor, is an important regulator of the pathway. Besides its role as a direct phosphatase of pAkt, PP2A also regulates the pathway by targeting S6K and PDK1 or by the interaction with MEK1 and AMPK, which crosstalk with the PI3K/Akt/mTOR pathway [12].

## 2. Consequences of PI3K/Akt/mTOR Perturbation

Because of the important roles that the PI3K/Akt/mTOR pathway plays in cellular physiology, pathologic alterations, which target the components or modulators of this pathway, could give rise to serious diseases. Indeed, mutations related to this pathway have been found in cancers with a high frequency [13]. Moreover, several viral agents and environmental toxicants also target this pathway and, in some cases, hijack its signaling for pathologic purposes [14,15]. Some physiologic products, such as free fatty acids, inflammatory factors and hormones, may also target this pathway detrimentally and contribute to various derangements, including cancer, T2D and hypertrophic cardiomyopathy [16,17]. Those reviewed viral agents, chemical toxicants and physiological products that target the PI3K/Akt/mTOR pathway are listed in Table 1. Note that the table does not include the apparent physical products, such as insulin and IGF.

**Table 1:** Substances that target the PI3K/Akt/mTOR pathway.

Viral Agents	Mechanism of Action	Reference
Mouse polyoma virus middle tumor antigen (PyMT)	▪ Associates with the plasma membrane and activates PI3K, resulting in PI3K/Akt/mTOR pathway activation	[18]
Mouse polyoma virus small tumor antigen (PyST)	▪ Inhibits PP2A, resulting in activation of Akt	[19]
Simian virus 40 small tumor antigen (SVST)	▪ Inhibits B56γ form of the PP2A holoenzyme, resulting in activation of Akt	[20]
Human papillomaviruses (HPVs) E6 protein	▪ Degrades TSC2, activating mTORC1 ▪ Activates PDK1 and mTORC2, activating Akt	[21] [22]
Human papillomaviruses (HPVs) E7 protein	▪ Inhibits PP2A, resulting in activation of Akt	[23]
Hepatitis C virus (HCV)	▪ Degrades TSC1/TSC2, activating mTORC1, which enhances negative feedback, inactivating the PI3K/Akt/mTOR pathway	[24,25]
**Chemical toxicants**	**Mechanism of Action**	**Reference**
Trivalent arsenic	▪ Induces ROS, inactivating Akt in lymphoma and leukemia cell lines ▪ Induces ROS, activating PI3K/Akt in prostate cancer cell line ▪ Activates JNK and induces mR-190 expression, activating Akt in a bronchial epithelial cell line	[26,27,28,29] [30] [31,32]
Cadmium	▪ Induces ROS, activating Akt in a bronchial epithelial cell line	[33]
Vanadate	▪ Induces ROS, activating PI3K/Akt in a prostate cancer cell line	[34]
Zinc oxide nanoparticles	▪ Induces ROS, inactivating the PI3K/Akt/mTOR pathway in mice peritoneal exudate	[35]
Superparamagnetic iron oxide nanoparticles (SPIONs)	▪ Activates Akt in a colon cancer cell line	[36]
Nicotine	▪ Activates PI3K through nicotinic acetylcholine receptors (nAChR)	[37]
NNK	▪ Activates PI3K nicotinic acetylcholine receptors (nAChR) ▪ Increases EGFR expression and Ras mutation	[37] [38,39]
Microcystin-LR	▪ Inhibits PP2A activity and decreases PTEN expression, activating PI3K/Akt in melanoma and colon cancer cell lines	[40,41]
**Physiological products**	**Mechanism of Action**	**Reference**
Free fatty acids (FFAs)	▪ Activates PKCθ, inhibitory phosphorylating IRS1 ▪ Upregulates PTEN, inactivating PI3K ▪ Induces ceramide, activating PP2A and inhibiting Akt ▪ Induces ceramide, activating Rheb/mTORC1/S6K; negative feedback attenuates Akt activation	[42,43,44] [45] [46] [47]
Interleukin (IL)-6	▪ Activates JNK1/2, inhibitory phosphorylating IRS1 ▪ Increases SOCS3 level, blocking IRS1 binding to IR ▪ Increases PTP1B activity, dephosphorylating IRS1 at tyrosine site	[48]
Tumor necrosis factor (TNF)-α	▪ Activates S6K, inhibitory phosphorylating IRS1	[49]
Angiotensin II and epinephrine	▪ Activate PI3Kγ through G-protein–coupled receptors	[50]

### 2.1. Genetic Alteration

Genetic alteration targeting the PI3K/Akt/mTOR pathway has been strongly implicated in cancer development. For example, genetic mutation or deletion causing somatic loss of the phosphatase and tensin homolog (*PTEN*) is the second most frequent oncogenic mutation of the human genome, eclipsed only by malignant mutations in *p53* [51]. It has been found in 60% to 80% of human prostate cancer and about 50% of melanoma, breast and endometrial cancers. PTEN is a direct antagonist of PI3K, serving as a control mechanism. Loss of *PTEN* activates the PI3K/Akt/mTOR pathway and is associated with both resistance to chemotherapy and reduced survival in human cancers [13]. Mutations of the *PIK3CA* gene, which encodes the p110α subunit of PI3Kα, have also been found in multiple human malignancies. Three frequent *PIK3CA* mutations, H1047R, E545K and E542K, all create an unhindered activation of Akt [13,52]. The mutation of all three isoforms of Akt, *AKT1/PKB*α, *AKT2/PKB*β and *AKT3/PKB*γ are also observed in multiple types of cancers [13,53]. Recently, 33 *MTOR* mutations were identified, which confer pathway hyperactivation, even under nutrient-starved conditions [54]. PP2A is critical in preventing malignant transformation of cells, because it maintains tight control of Akt and other oncoproteins [55]. Mutations or other disruptions of PP2A activity, with the resultant loss of Akt control, are oncogenic. Although mutations of PP2A are found at a low frequency [56], more common mutations of *p53* prompt the expression of CIP2A, which inhibits PP2A, thus leading to malignancy [57]. CIP2A overexpression has been found in over 70% of acute myeloid leukemia (AML), ovarian, prostate, lung, colon and gastric cancers, as well as squamous cell carcinomas of the head and neck (HNSCC) [57,58]. Indeed, genetic alterations causing summary activation of the PI3K/Akt/mTOR pathway potentiate both survival and proliferation, even in the absence of growth factors. This unbridled cell growth and survival is the hallmark of malignancy. Therefore, some hypothesize that cancers may suffer from an oncogenic “addiction” to the constant activation of the PI3K/Akt/mTOR pathway [59].

### 2.2. Viral Usurpation

Because of the crucial regulatory role of the PI3K/Akt/mTOR pathway in protein translation and cell survival, many DNA and RNA viruses target and commandeer this pathway for the purpose of successful replication [60]. Viral activation of the PI3K/Akt/mTOR pathway prompts protein translation and macromolecular synthesis, which is critical for viral replication and survival [61,62]. Moreover, the activated pathway suppresses various cellular stress and protection responses, so that viral replication can proceed unimpeded. For example, viral infection often disturbs calcium homeostasis. As one of the cell’s self-protection mechanisms, the disturbed calcium homeostasis would normally inhibit mTORC1 through TSC2 and consequently halt protein translation [63]. Viruses overcome this mechanism by sustained activation of the PI3K/Akt/mTOR pathway, regardless of calcium homeostasis. The hijacked pathway maintains robust protein synthesis and fosters viral replication [61]. Other viral-induced cell stress, including hypoxia, energy deprivation and amino acid deprivation, also normally decreases the activity of the PI3K/Akt/mTOR pathway. Viruses often counteract this for their own benefit [61].

Viruses activate the PI3K/Akt/mTOR pathway at various sites and with different behaviors, which are reviewed by Buchkovich *et al.* [61] and Diehl *et al.* [62]. Viral alteration of the pathway leads to concerning biological toxicities, in addition to other virus-specific pathogenesis. In this review, we mainly focus on the metabolic disruption and oncogenesis induced by viral perturbation of the PI3K/Akt/mTOR pathway.

Amongst a number of types of DNA viruses, polyomaviruses have been found to promote oncogenesis. The most studied polyomaviruses are mouse polyoma (Py) virus, simian virus 40 (SV40) and the papillomaviruses. All three types of polyomaviruses have been shown to target the PI3K/Akt/mTOR pathway, contributing to oncogenesis [61]. Differential early gene splicing of the Py virus produces three early proteins: large tumor antigen (PyLT), middle tumor antigen (PyMT) and small tumor antigen (PyST). Among them, PyMT is a potent oncoprotein [61]. Studies have shown that PyMT can associate with the plasma membrane and activate PI3K, thereby hijacking the PI3K/Akt/mTOR pathway [18]. It has also been reported that PyST can activate the pathway by directly inhibiting PP2A [19]. The early region of the SV40 gene encodes two oncoproteins: the large tumor (SVLT) and small tumor (SVST) antigens. The SVLT binds to p53 and retinoblastoma (Rb) family proteins to induce cell transformation, in a process that is dependent on IRS1 signaling [64]. In contrast, SVST specifically inhibits the B56γ form of the PP2A holoenzyme, resulting in over-activation of Akt signaling. SVST also inhibits p53 and causes accumulation of oncoprotein c-Myc. These combined SVST effects together may lead to cell transformation [20].

Human papillomaviruses (HPVs) have a high risk of causing cervical cancer and are associated with head and neck malignancies [22,65]. Two of the HPV early proteins, E6 and E7, are highly associated with oncogenesis. The E6 protein can degrade TSC2, an inhibitor of mTORC1 [21]. It can also activate Akt by targeting its activators, PDK1 and mTORC2 [22]. The E7 protein targets PP2A, decreasing its inhibitory phosphatase activity, thereby maintaining the activation of Akt [23]. Taken alone, the viral-activated PI3K/Akt/mTOR pathway strongly promotes protein synthesis. However, when combined with p53 and Rb inactivation, the activated pathway could potentially become oncogenic [61].

Hepatitis C virus (HCV) is a small, positive-sense, single-stranded RNA virus. It has long been observed that infection with HCV increases steatohepatitis, which in turn is associated with T2D [15]. Further, because the PI3K/Akt/mTOR pathway is a major controller of cellular translation and macromolecular synthesis, it has been suspected that HCV would target and activate this pathway for the benefit of viral protein synthesis. This hypothesis was validated when studies delineated HCV targeting TSC1/TSC2, the negative regulator of mTOR. By increasing the degradation of TSC1/TSC2, HCV promotes mTOR and S6K expression, resulting in the activation of translation and protein synthesis [24]. Meanwhile, the activation of mTOR/S6K also enhances the negative feedback signaling to IRS1, resulting in insulin resistance in hepatocytes [25].

### 2.3. Chemical Toxicants

Chemical toxicants, such as heavy metal compounds and natural peptides, can exert toxicological effects by targeting the PI3K/Akt/mTOR pathway [14]. Compounds, including arsenic, cadmium, mercury, vanadate, nicotine, superparamagnetic iron oxide nanoparticles and zinc oxide nanoparticles, can cause either apoptosis or malignant transformation, depending on the compound and experimental conditions [30,33,34,35,36,37,66,67].

For example, inorganic trivalent arsenic (As^3+^) is an environmental toxicant and carcinogen. As^3+^ exposure poses risks for human malignancies of the skin, the hematopoietic system, the respiratory tract and urinary bladder [66]. Paradoxically, As^3+^ also possesses a potent ability to induce apoptosis and is used as an anticancer drug, especially in the treatment of acute promyelocytic leukemia [68]. Since carcinogenesis and apoptosis are both tightly linked to the PI3K/Akt/mTOR pathway, it could be expected that As^3+^ would differentially target the pathway to accomplish these opposite effects on cell fate.

Studies show that arsenic trioxide (As_2_O_3_) induces apoptosis with relatively high concentrations (usually above 5 μM) and over 24 h of treatment in multiple cell lines, such as human Burkitt’s lymphoma cell line (Ramos) and human leukemia cell lines (U937, NB4 and K562) [26,27,28,29]. Further, mechanistic studies reveal that such treatments induce reactive oxygen species (ROS), which decrease Akt activity, resulting in an increase in pro-apoptotic signals, including activation of BAD and FoxO3a, reduced mitochondrial transmembrane potential, release of cytochrome c and activation of the caspase family, all culminating eventually in cell death [26,27]. These pro-apoptotic features of arsenic prompt its use in the clinical setting, especially in the treatment of acute promyelocytic leukemia (APL) [26]. However, there is also a concern that arsenic usage may provoke cardiotoxicity, altering cardiac conduction, as demonstrated on electrocardiograms as ST-T wave changes and QT interval prolongation [69]. Recent studies have found that activation of the PI3K/Akt/mTOR pathway may protect the cardiomyocyte from such alteration and promote cell survival [70,71].

Instead of suppressing Akt signals and inducing cell death, As^3+^ was found to activate Akt, through JNK signaling crosstalk or microRNA miR-190-related regulation, in a human bronchial epithelial cell line, BEAS-2B. The activated Akt leads to an increase in proliferation and migratory ability, suggesting the role of As^3+^ in cell transformation [31,32]. As^3+^ has also been shown to activate Akt in mouse epidermal Cl41 cells, when treated with 0.1 to 10 μM for 1 h. Moreover, a longer time (over 72 h) and lower concentration (1.25 μM) exposure to arsenite increases the Cl41 cells’ proliferation and the portion of the cells in S-phase, due to cyclin D1 expression caused by the PI3K/Akt/mTOR pathway activation [66]. Moreover, arsenite activates Akt through ROS, leading to the increase of both hypoxia-inducible factor 1 (HIF-1) and vascular endothelial growth factor (VEGF) protein levels in human prostate cancer cell line DU145, thus contributing to carcinogenesis [30]. This pattern of metal toxicants targeting the PI3K/Akt/mTOR pathway to stimulate HIF-1 and VEGF expression has also been found in cadmium and vanadate treatment [33,34]. Notably, these metal compounds usually target the PI3K or Akt indirectly through ROS, which also causes genome stress. Thus, the survival signals from the PI3K/Akt/mTOR pathway in the milieu of DNA damage should be considered an important oncogenic mechanism in chemical toxicity.

Nanoparticles, such as zinc oxide nanoparticles and superparamagnetic iron oxide nanoparticles (SPIONs), can alter cell fate through perturbation of the PI3K/Akt/mTOR pathway. Zinc oxide nanoparticles induce ROS generation, which further inactivates the PI3K/Akt/mTOR pathway, leading to upregulation of autophagy marker LC3, activation of caspase 3 and apoptosis in primary culture of cells from mice peritoneal exudate [35]. SPIONs, however, can activate Akt in a human colon cancer cell line, HCT116, and increase HCT116 cell proliferation [36].

Another well-studied example of toxicants targeting the PI3K/Akt/mTOR pathway for carcinogenesis lies in tobacco components activating this pathway to induce lung cancer. Nicotine and 4-(methylnitrosamino)-1-(3-pyridyl)-1-butanone (NNK) are two major tobacco carcinogens shown to activate the PI3K/Akt/mTOR pathway [37]. Studies show that nicotine and NNK are potent agonists of nicotinic acetylcholine receptors (nAChR), which are expressed in lung epithelial cells and can activate PI3K [37]. Moreover, NNK can also activate PI3K by increasing the expression of EGFR and by induction of Ras mutation, which is found in about 25% of smoking-related human lung adenocarcinomas [38,39]. The activation of the pathway is PI3K-dependent, since the PI3K inhibitor LY294002 can abolish the activation of Akt by nicotine and NNK [37]. Indeed, the activation of the whole PI3K/Akt/mTOR pathway, from PI3K to S6K and 4E-BP1, has been verified by extensive studies [37,72] and results in the inhibition of pro-apoptotic proteins Bad and Bax, the induction of pro-survival protein survivin and the activation of NF-κB [72,73,74]. Notably, the NNK metabolites can also bind to DNA and cause gene mutations, including *TP53* and *KRAS* [75]. Thus, similar to the metal compounds, tobacco components may induce malignancy by combining DNA damage with overriding survival signals exerted by PI3K/Akt/mTOR pathway activation [76].

Toxicants that primarily target the modulators of the PI3K/Akt/mTOR pathway, such as PP2A, can also exert their toxicity through this pathway, although the functions of this pathway may be opposed by other mechanisms. For example, microcystin-LR, a cyanobacterial peptide toxin found in toxic algae blooms, directly binds to the catalytic subunit of PP2A and inhibits its phosphatase activity. The consequential activation of Akt is associated with cell transformation [40,41]. However, when the cells are exposed to higher concentrations of microcystin-LR, the loss of PP2A activity causes activation and accumulation of p53 protein and ceramide, which overcomes the pro-survival signal from Akt and induces cell death [77,78,79]. These findings also suggest that the pro-survival functions of Akt activation could be counteracted by other mechanisms.

It is also noteworthy that the toxicity exerted by environmental toxins or chemical compounds on the cellular pathways are usually nonspecific. Other pathways alongside PI3K/Akt/mTOR may also be targeted and contribute to the toxicity. For example, the MAPK and NF-κB pathways play important roles in the apoptosis or transformation caused by multiple toxicants, such as arsenite, cadmium, carbon nanotubes and polybrominated diphenyl ether (PBDE) [33,80,81,82,83,84]. Nicotine and NNK also activate protein kinase C (PKC), the ERK MAPK pathway and the janus-activated kinase/signal transducer and activator of transcription (JAK/STAT) pathways for lung tumorigenesis [85]. The complexity of the numerous signaling pathways involved makes it a formidable task to build computational models for the toxicity analysis. Nonetheless, the patterns of the alteration of individual signaling pathways, which have been associated with cell fate alteration, could help elucidate the cellular signaling mechanisms for therapeutic reference. Indeed, based on the material that we have reviewed, in most cases when the compound induces apoptosis, it was associated with inhibition of the PI3K/Akt/mTOR signaling pathway and especially suppression of Akt activity. In contrast, the activation of the pathway was overwhelmingly associated with survival or malignant transformation [14,33,86,87]. Thus, the PI3K/Akt/mTOR pathway is intimately involved in cell fate determination and therefore an important target for therapeutic purposes.

### 2.4. Physiological Products

The last 20 years have witnessed the escalation of many diseases, including diabetes, obesity, cancer and cardiovascular diseases. Interestingly, these diseases are related to the deregulation of the PI3K/Akt/mTOR pathway. In particular, the deregulation of the pathway is a key feature of cancer and T2D [88]. In contrast to the oncogenic hyper-activation of the PI3K/Akt/mTOR pathway, it is the hypo-activation of this pathway that attenuates cellular insulin response, causing insulin resistance and even T2D [89]. While genetic causes of T2D are less frequently seen, physiological causative factors, including free fatty acids (FFAs) and inflammatory factors, target the PI3K/Akt/mTOR pathway and interfere with the insulin response [16]. Moreover, physiological hormones like IGF-1 and angiotensin II also target the PI3K/Akt/mTOR pathway, resulting in either physiological, or pathological, growth of the heart [90].

High concentrations of circulating FFAs are tightly associated with obesity and T2D. With worsening obesity, it becomes more difficult for adipose tissues to contain fat. Consequently, the concentration of FFAs increase in blood. Multiple components of the PI3K/Akt/mTOR pathway are targeted by FFAs. Studies show that FFAs activate PKCθ, which phosphorylates IRS1 at Ser^1101^ and thereby blocks IRS1 tyrosine phosphorylation and further signaling via PI3K [42,43,44]. FFAs also attenuate the insulin activation of Akt through upregulation of PTEN [45]; or through induction of ceramide, which, in turn, activates PP2A and thereby decreases Akt activation [46]; or by enhancing the mTOR/S6K to IRS1 negative feedback signaling [47]. Each of these culminates in insulin resistance.

Elevated plasma cytokines, including TNFα and IL-6, were detected in obese patients and have been shown to promote insulin resistance. Experimental data show that extended (24 h) pretreatment of IL-6 impaired the activation of Akt by insulin [48], whereas TNFα impaired the insulin signaling at IRS1, through activating S6K-related negative feedback signaling [49], both contributing to insulin resistance. A recent revelation shows that FGF1 can, both acutely or chronically, sensitize diabetic mice to insulin and increase glucose uptake in an insulin-dependent manner [91]. It was shown in this study that FGF1 functions through activation of Akt signaling [91]. It remains to be determined whether the insulin sensitization seen with FGF1 is due to an enhancement of pAkt-IRS1 positive feedback.

Due to its crucial role in cell growth, the PI3K/Akt/mTOR pathway has been found to regulate the size of cardiomyocytes in both the developmental stage and in adulthood [92]. Moreover, a *PTEN* mutation-induced deregulation of the PI3K/Akt/mTOR signaling pathway was associated with hypertrophic cardiomyopathy [93]. There are two types of cardiac hypertrophy in the adult heart: physiological cardiac hypertrophy and pathological cardiac hypertrophy, which is also known as hypertrophic cardiomyopathy [94]. Physiological cardiac hypertrophy is considered adaption to exercise training and contributes to enhanced cardiac function [94]. In the process of physical exercise, the myocardium produces IGF-1, which activates the PI3K/Akt/mTOR signaling pathway in cardiomyocytes and promotes physiological growth of the myocardium [95,96]. Further studies found that PI3Kα, a specific subtype of the PI3K family, is critical for physiological exercise-induced heart growth, but is not associated with pathologic cardiac hypertrophy [97,98]. Overexpression of PI3Kα in the heart can even be protective against pressure overload, whereas a PI3Kα dominant-negative model shows an absence of a physiological hypertrophic response following exercise and susceptibility to the weak, over-expanded, thin-walled chambers found in dilated cardiomyopathy [90]. In contrast to physical exercise, cardiac stress such as excessive vascular afterload, induces a pathological cardiac hypertrophy, with a thickening and stiffening of the chambers, progressively reducing pump efficiency (specifically, the ejection fraction) and leading to “heart failure”, which is the inability of the heart to perfuse sufficiently to meet the body’s metabolic needs [94]. Studies show that cardiac stress induces the generation of neurohormones, such as angiotensin II and epinephrine, which specifically target PI3Kγ, another subtype of the PI3K family, through G-protein-coupled receptors (GPCRs) [50,90]. It is so far understood that the prolonged activation of PI3Kγ signaling, coupled with other pathways, such as MAPK, leads to hypertrophic cardiomyopathy [90,94,99].

## 3. The PI3K/Akt/mTOR Pathway in Molecular Targeted Therapy: Progress and Worry

From the evidence reviewed, it may be generally summarized that the alteration of the PI3K/Akt/mTOR pathway and the consequential diseases are closely related to the pro-growth and pro-survival functions of the pathway. When the pathway is activated without control, the resulting hyper-survival and proliferation of the cells contribute to cancers and chemotherapy resistance. Conversely, hypo-activation of the same pathway hinders its response to stimulus hormones. In particular, if the stimulus hormone is insulin, then T2D may result. The detailed elucidation of the pathway by researchers now provides important targets for disease treatment.

Great progress has been made in the field of molecular targeted cancer therapy during the last ten years, with a number of candidate therapeutic targets identified [100]. The over-activated PI3K/Akt/mTOR pathway, because of its essential role in promoting cancer growth and survival, is deemed one of the most promising signaling pathways to target for therapeutic intervention [13,101]. Indeed, the rationale for the inhibition of the PI3K/Akt/mTOR pathway in targeted cancer therapy is compelling and is supported by four major elements of clinical evidence: the activation of the pathway is common in multiple cancers; its activation is found throughout carcinogenesis; and its activation is a poor prognostic factor and may also confer resistance to chemotherapy [102]. Furthermore, there has been conjecture that cancer cells rely on the activation of the PI3K/Akt/mTOR pathway for survival, because of the adverse conditions in which they grow, such as acidic or hypoxic conditions. Healthy cells, in contrast, do not rely as heavily on the pathway and are thus less vulnerable to cell death when the pathway is inhibited [102]. This is important, because it suggests that targeted inhibition of this pathway would harm malignant cells and relatively spare healthy cells. Inhibition of certain components of the PI3K/Akt/mTOR pathway may slow down or even stop cancer growth or sensitize cancer cells to chemotherapy.

Due to this sound rationale, much effort across academia and industry has been vigorously focused on the development of such molecular targeted therapies. Thus far, more than 50 drugs inhibiting the PI3K/Akt/mTOR pathway are at different stages of development, with 30 such inhibitors reaching clinical trials [103]. Analogues of rapamycin are currently in the lead, having already been approved by the United States Food and Drug Administration (FDA) for the treatment of renal carcinoma, neuroendocrine tumors and mantle cell lymphoma [104]. The pharmacodynamics of these drugs have been extensively studied and reviewed elsewhere [103,105,106,107]. However, it is important to note that the results of some recent early clinical trials have been underwhelming compared to the high expectation from preclinical studies, thus raising urgent calls for the improvement of therapeutic strategies [103,104].

One of the potential problems with inhibiting the PI3K/Akt/mTOR pathway is that the pathway remains an important part of healthy cell metabolism, and as elucidated previously, inhibition of this pathway in healthy cells can lead to decreased insulin sensitivity and potentially T2D [108]. Therefore, one of the great challenges in cancer therapy with the inhibitors of PI3K/Akt/mTOR would be the subsequent development of hyperglycemia, hyperlipidemia and other metabolic disorders.

Indeed, clinical trials found that temsirolimus and everolimus, which are analogues of rapamycin, caused hyperglycemia in 26% and 50% of patients, respectively, as well as 11% and 12% Grade 3–4 toxicity [109]. Similarly, 37% of patients experienced hyperglycemia when treated with BKM120, a pan-class I PI3K inhibitor, with 9% Grade 3–4 toxicity [105]. Acute hyperglycemia has also been found with Akt inhibitor treatments [110]. These adverse metabolic effects suggest that inhibitors of the PI3K/Akt/mTOR pathway contribute to insulin resistance in normal cells.

In a more sophisticated manner, treatment with rapamycin and its analogues caused an uprising of interferon gamma (IFNγ) due to a perturbation of the immune system [111]. On the one hand, the cytokines may help kill cancer cells [111]. On the other hand, it is worrisome that these cytokines interfere with the insulin response in normal cells [112]. The interferon cytokines, along with their risks of hyperglycemia and hyperlipidemia, may also cause apoptosis of pancreatic β cells and eventual pancreatic endocrine failure, and the T2D patient would need to start insulin therapy [113]. Together, these adverse drug effects effectively limit the dose and duration of these otherwise promising drugs.

It is important to note that to maintain malignancy remission, the PI3K/Akt/mTOR kinase inhibitors are often life-long therapies; otherwise, the reactivation of the pathway would cause the re-emergence of malignancy [114,115,116,117]. Therefore, aside from some common adverse effects caused by PI3K/Akt/mTOR inhibitors, such as hyperglycemia, rash, asthenia and mucositis, there are other chronic adverse effects that might develop over a longer term and that might not be detected in initial clinical trials. One of the concerns is that PI3K inhibitors may pose a threat to heart health, because PI3K signaling is important for physiologic cardiac muscle growth and response to stress. Impaired PI3K function could render the myocardium vulnerable, even to the stress of moderate physical exercise, thus increasing the risk of heart failure [114].

In addition to the toxic effects exerted by the PI3K/Akt/mTOR-targeted inhibitors described above, there are regulatory feedback and drug resistance issues that also diminish the therapeutic effect of these drugs [10,118,119]. For instance, while rapamycin and some analogues inhibit the activity of mTORC1 and decrease the activity of S6K and 4EBP-1/eIF4E for the purpose of halting protein translation, the negative feedback (S6K inhibiting IRS1) is also diminished. This could sensitize growth factor signaling through the IRS and thus perversely strengthen pro-survival effects in the cancer cells by overriding PI3K and Akt [120,121]. Another example [122] is found in newer forms of mTOR kinase inhibitors, which block both the activity of mTORC1 and mTORC2, aiming to not only reduce the mTORC1 activity, but also to block the phosphorylation of Akt at Ser^473^ by mTORC2. Therefore, it was hoped that these mTOR kinase inhibitors could avoid Akt activation due to mTORC1 inhibition-related feedback. However, experimental data found that the inactivation of Akt by such treatments is only transient. Moreover, the transient inhibition of Akt activity could activate a FoxO-related feedback, resulting in upregulation of receptor tyrosine kinase (RTK) expression and further sensitizing the malignant cells to multiple growth factors [122]. One exception to this has been mTOR inhibition with metformin, which employs a different strategy to inhibit mTORC1. It is well established that metformin activates AMPK, which inhibits mTORC1 activity. Moreover, activated AMPK also directly phosphorylates IRS1 at Ser^789^ and, thus, decreases the activation of Akt [123]. It is certainly interesting that metformin can not only treat the insulin resistance of T2D, but also may be effective for some malignancies [124].

Compared with traditional therapies that have less directed modes of action (e.g., chemo- and radio-therapy), molecular targeted therapy is a great advancement in therapeutics, because aberrant molecular signaling is the key to the pathogenesis of these diseases. However, molecules in a cell do not function in isolation; it is the entire network of interactions between the molecules that determines system-level regulatory mechanisms. Indiscriminately targeting individual molecules in a biomolecular network, without an understanding of the regulatory mechanisms in which they participate, could give rise to unexpected and highly undesirable results [1].

Indeed, as promising as molecular targeted therapy seems, the current state of drug development is facing great challenges. The toxic side effects and drug resistance obstacles encountered greatly limit the use of single agents as monotherapies to target the PI3K/Akt/mTOR pathway in cancer treatment [104]. A more promising approach seems to be to target the PI3K/Akt/mTOR pathway at multiple sites, as well as along related pathways, as a means to maximize efficacy, while reducing side effects; it may also be possible to identify inhibitors that target aberrant PI3K/Akt/mTOR pathways exclusively within cancer cells.

## 4. Computational Modeling for Therapeutic Guidance

We have reviewed a wide array of agents perturbing the PI3K/Akt/mTOR pathway (viruses, gene mutations, chemical toxicants, physiologic products, *etc.*). The great diversity of these agents notwithstanding, the responses of the pathway share common features and consequences, as we have demonstrated. This implies that the pathway possesses regulatory mechanisms that are inherent to the pathway itself, which can be revealed by studying the dynamics of the pathway, without referring to the identity of the initial perturbation. A thorough understanding of these regulatory mechanisms is important for the testing of drugs. In combination therapy, an optimally proportionated drug regimen is desired so as to obtain the maximum synergistic effect. However, selecting the appropriate biologically active dose, both for single agent and combination therapy, remains a conundrum [103]. The regulatory mechanisms, if revealed, can give direct guidance for producing a low dosage combination therapy with minimal toxicity and drug resistance.

Because the regulatory mechanisms involve molecular interactions of the entire pathway, mathematical modeling and *in silico* studies are indispensable to obtain a holistic grasp of the system-level properties. On the other hand, the high costs associated with *in vitro* and *in vivo* research, as well as the emergence of quantifiable biomarker data on these pathways, has prompted study of biological processes *in silico* [125,126]. Thereby, the urgent needs of optimization in therapeutic strategies, the accumulation of experimental evidence and the advancement of *in silico* studies have given rise to the exploration of computer-aided targeting.

Introduction to computer-aided targeting:

(a) Computer-aided targeting in this review refers to the execution of *in silico* studies that simulate the dynamic changes of certain signaling pathways and using the gleaned information to guide experimental or clinical modulation of the pathway. Such dynamic changes may range from the system level, which refers to the regulatory network of the pathway, to the molecular level, which refers to the structure and conformation of a specific molecule. Guidance gleaned from these computational studies is potentially useful in many facets of therapy, including the formulation of optimized drug combinations to maximize synergistic efficacy with reduced toxicity and designing pharmaceuticals to preferentially target one specific type of molecule to prevent off-target toxicity.
(b) Adequate *ex silico* data, such as abundant protein-protein interaction evidence or nuclear magnetic resonance (NMR) and X-ray crystallography-illustrated protein structures are necessary for realistic computerized simulation. The methods employed in computer-aided targeting include a collection of software-based modeling applications, each crafted to simulate different levels of pathway and molecular interaction.
(c) For example, in order to formulate an optimized composition of kinase inhibitors in combined therapy, a thorough understanding of the dynamic flow of the signaling pathway should first be acquired from real-world experimental evidence. Then, based on this descriptive experimental data, nodal points (nodes) along the pathway can be defined, and a complicated network may be simplified to a backbone framework for system-level *in silico* simulation. Mathematical modeling, harnessing prominent biological equations, such as the Michaelis–Menten equation (modeling enzyme kinetics), the Goldbeter–Koshland equation (modeling biological states impacted by both promoting and inhibiting enzymes) and the law of mass action (modeling the behavior of solutions in dynamic equilibrium) can then be used to depict the mechanistic flow of the framework. Graphic output from mathematical models are generated, and the values of different nodes may then be manipulated to mimic different biological states. A review of this graphic output often reveals boundaries formed by combinations of nodal states, highlighting interesting and useful cases of signaling dynamics. Finally, comparing the nodal configurations of pathological states to those of normal states may identify numerous points of transition between the two; analysis of these can identify the most efficient transition from pathological to normal, and this represents an optimized therapeutic strategy.
(d) *In silico* simulations at the molecular level can also provide guidance on drug design. For example, based on NMR or X-ray crystallographic data, molecular dynamics (MD) simulation applies classical and quantum mechanics to calculate the forces between bonded and non-bonded atoms and, from this, builds functionally useful simulations of whole molecules, especially the receptors of interest as they function and move in three-dimensional space. Powerful software packages, such as AMBER, CHARMM and NAMD, allow sophisticated MD simulations to be achieved. MD simulation can reveal subtle dynamic differences between a targeted molecule and other closely related ones. Furthermore, software packages like FTMAP can be applied to computationally manipulate ligand probes on multiple simulated protein conformations, in order to identify cryptic and allosteric binding sites which might otherwise be easily overlooked by structure-based drug design. Moreover, docking programs, which employ a method known as the relaxed complex scheme (RCS), can be applied to virtually screen the binding sites of drug candidates in a simulated flexible receptor; free-energy calculations can predict the drug-receptor binding efficiency; and techniques, such as alchemical techniques, can greatly simplify the computation. These docking and free-energy analyses are thus particularly advantageous to discover drugs with high specificity to the targeted protein.

A mathematical model in the form of coupled ordinary differential equations was previously defined, in order to study a broad pathway in the context of insulin signaling [127]. The mechanistic model described the insulin stimulated signaling dynamics, including IRS1 phosphorylation and the following activation of PI3K and Akt, as well as GLUT4 translocation and the pAkt-IRS1 positive feedback loop. Moreover, differential equations were formed with rate constants and parameters informed by previous experimental data [127]. The model was used to simulate the detailed dynamics of insulin signaling. However, it does not serve the purpose of unveiling the simple and general mechanisms that underlie pathway regulation under normal and pathologic conditions.

**Figure 2:**
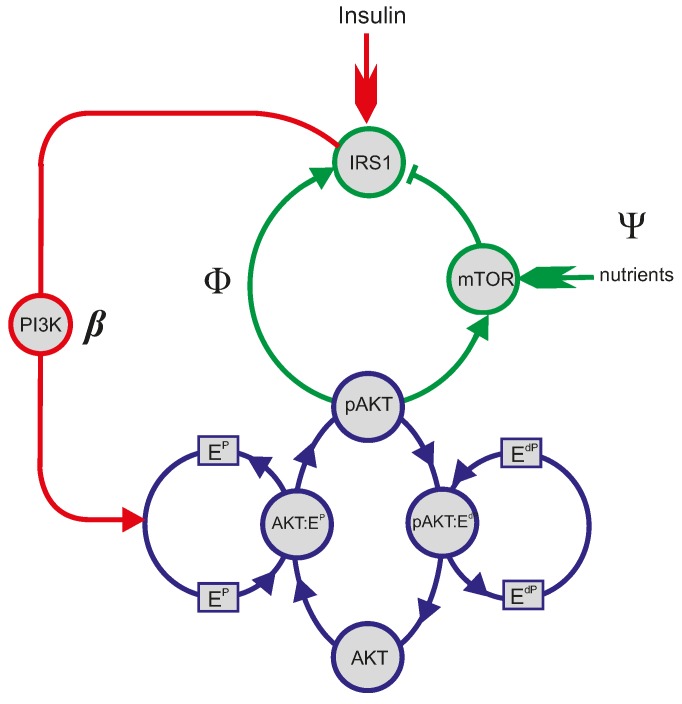
The backbone structure of the PI3K/AKT/mTOR pathway. The pathway can be divided into three components: the input (colored in red), the output (colored in blue) and the feedback (colored in green). E^P^ includes PDK1 and mTORC2; E^dP^ includes PP2A and PHLPP1/2.

To reveal such regulatory mechanisms, the backbone structure of the PI3K/Akt/mTOR pathway must first be obtained to filter out unimportant information. Currently, there exist sufficient data to evaluate the backbone structure, on the basis of which a simplified mathematical model can be built to realistically simulate both the normal function and the pathologic alterations of the pathway. Such a model may be useful to test strategies to rectify a deranged pathway. Our previous studies have constructed such a backbone network and its computational model to facilitate the systematic study of the PI3K/Akt/mTOR pathway [89,128]. The simplification was crafted according to the following considerations: First, since Akt is the master regulator of the pathway, the detailed phosphorylation and dephosphorylation cycle (PdPC) of Akt was central to, and therefore largely retained in, the backbone structure. Second, molecules along a linear cascade were represented by only three molecules: two ending ones and a middle one. These simplifications led to a backbone network structure as shown in Figure 2, which includes the PdPC of Akt, the positive feedback loop and the negative feedback loop. In the PdPC, E^P^ groups the protein kinases, PDK1 and mTORC2; E^dP^ groups the phosphatases, PP2A and PHLPP1/2. With this simplified model, the regulatory mechanism of the pathway can be represented by the response curve *A*(*I*), where *I* represents the concentration of the growth factor (e.g., insulin), and *A* = [pAkt]/[Akt]_total_ is the percentage of phospho-Akt.

The regulatory mechanisms may reflect key features of physiologic/pathologic phenotypes. That is, the shape of the response curve may contain information about the pathway functioning: a normal response may correspond to one kind of shape, and a pathologic response may correspond to another. Of course, such a mathematical model cannot be expected to reveal all of the complexities of diseases or normal functions, but the insights gained are valuable and may provide crucial insights into further investigations. This is especially true when one only considers the latency phase of diseases, during which non-essential body reactions do not happen.

### 4.1. Mathematical Analysis of the PI3K/Akt/mTOR Pathway

With the simplified model, regulatory mechanisms, as visualized in the resultant response curves, can be discovered *in silico* through numerical simulation. One first assigns a set of parameter values to the model and then runs the model to obtain a response curve *A*(*I*). By sampling of the parameter space for sufficiently long, one can obtain a broad canvas of the biologically relevant responses with certain confidence. However, because the search depends on sampling and the search size scales exponentially with the number of parameters, it cannot be guaranteed that all phenotypes will be found. More importantly, simulation studies without analysis do not readily reveal system-level regulatory mechanisms or illuminate connections between various phenotypes.

To overcome this difficulty, a novel approach was developed to analyze the pathway [89,128], which significantly reduced the computational complexity. Because a response curve is ultimately determined by the parameters of the mathematical model, the space of parameters can be divided into many regions, each corresponding to a phenotype. The boundaries between the regions (which separate the phenotypes) can be identified by the singularity theory, which is specialized for the detection of qualitative changes. As an analytical approach, singularity analysis does not depend on extensive simulations.

Mathematical analysis revealed that the parameter space of the pathway can be well characterized. Although there are many parameters in the model, it was found that several parameters always group together to form a composite parameter, assuming that the system is in the steady state. In total, there are only four such composite parameters: β, α, *K^P^* and *K^dP^*. The parameter β positively correlates with E^P^/E^dP^. Assuming E^dP^ is a constant, then β positively correlates with E^P^, which, in turn, positively correlates with the strength of the edge IRS1 → E^P^ (e.g., an increase in PI3K activity can increase E^P^ and, hence, β). The parameter α = φ − ψ represents the difference between φ (which is proportional to the strength of positive feedback pAkt-IRS1) and ψ (which is proportional to the strength of negative feedback pAkt-mTORC1-S6K-IRS1). The parameters:

(1) $$K^{P}=K_{m}^{P}/{\left[\text{Akt}\right]}_{total}$$

and:

(2) $$K^{dP}=K_{m}^{dP}/{\left[\text{Akt}\right]}_{total}$$

are the Michaelis constants of phosphorylation and dephosphorylation kinetics. By further assuming the symmetry of PdPC (*i.e.*, *K^P^* = *K^dP^* = *K*), the number of parameters reduce to three: β, α and *K*.

In order for the system to have fine dynamical properties (sensitivity, robustness, adaptivity), *K* should not be too large. According to numerical simulations, it was found that *K* ≤ 0.05 provided sufficient resolution [89]. This condition may well be realistic, because Akt is an abundant protein. An intriguing future direction would be to determine the values of *K^P^* and *K^dP^* by biochemical experiments and to test their values. In the following, we assume that *K* is indeed small.

**Figure 3:**
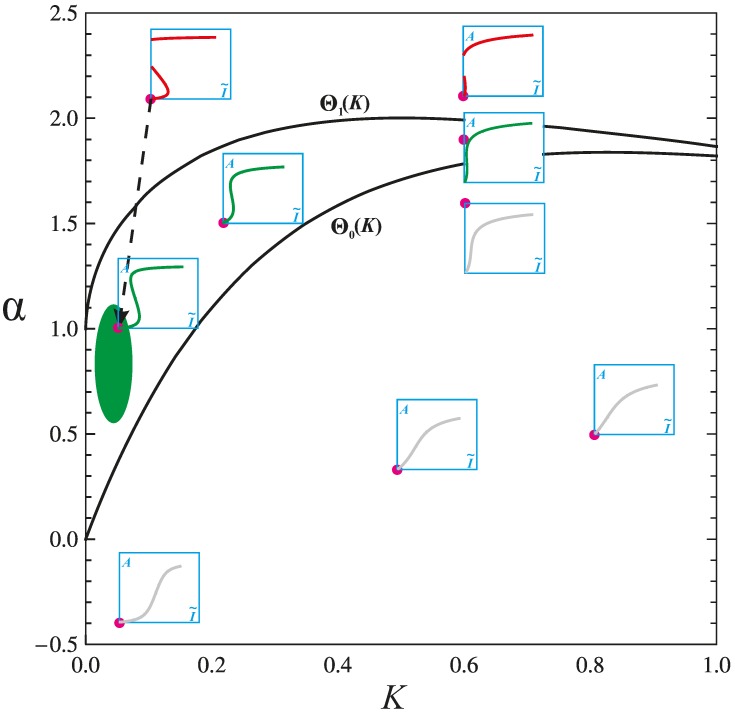
The phase diagram *K versus* α, categorizing different shapes of the pathway’s response curve. The parameter *K* is the Michaelis constant divided by the total Akt concentration. The parameter α corresponds to the net strength of positive feedback over negative feedback. The two functions Θ_0_(*K*) and Θ_1_(*K*) trace out two curves that divide the plane into three regions.

**Figure 4:**
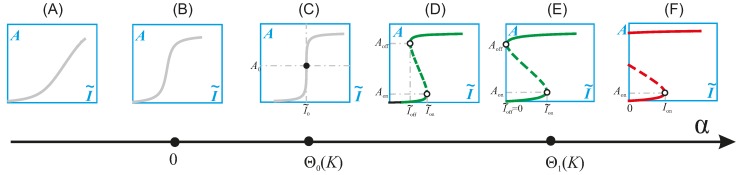
The deformation of the response curve as the parameter α changes, when K_1_ = K_2_ = 0.01 and β = 1, are fixed. (**A**) The monotone type with low sensitivity; (**B**) the monotone type with high sensitivity; (**C**) the vertical tangent type when α = Θ_0_(0.01); (**D**) the toggle switch; (**E**) the threshold of the irreversible switch when α = Θ_1_(0.01); (**F**) the irreversible switch.

By fixing β, the parameter space reduces to the *K* ~ α plane. Figure 3 corresponds to the case β = 1. By applying singularity analysis, Wang identified two singularity boundaries that divide the plane into three regions [89]. For any two points within the same region, their corresponding response curves are qualitatively the same (although they are, of course, different). For any two points in different regions, their response curves are qualitatively different. The three regions, from the top down, are the regions of the irreversible switch, the toggle switch and the monotone type, respectively. Following the assumption that *K* is small, the change of the shape of the response curve is actually driven by the change of α. As α increases, the response curve gradually changes from the monotone type to the toggle switch and, finally, to the irreversible switch (Figure 4, which is obtained with *K* = 0.01).

**Figure 5:**
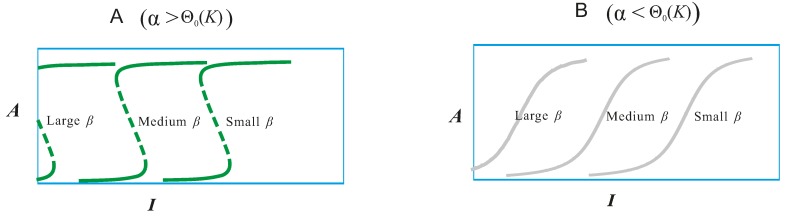
The shift of the response curve *A*(*I*) along the *I*-axis by altering β only. The shift is almost parallel, because the shape of the response curve is very insensitive to the value of β. (**A**) The response curve is a toggle switch (α > Θ_0_(*K*)); (**B**) The response curve belongs to the monotone type (α < Θ_0_(*K*)).

The above narratives are based on the case β = 1. What would happen if β changes? Mathematical analysis revealed that β causes a shift of the response curve along the *I-*axis [1,129]. The shift is approximately parallel, especially when *K* is small. As β decreases, the response curve makes an almost perfect shift to the right, which holds true for both the case α > Θ_0_(*K*) and α < Θ_0_(*K*) (Figure 5). Note that when *K* is small, one has Θ_0_(*K*) ≈ 0. For the limit case *K* → 0, one has Θ_0_(*K*) → 0, and the parallelism of the shift becomes exact.

Finally, it is worth mentioning that the revealed regulatory mechanisms are general. The PI3K/Akt/TOR pathway, ubiquitously present in almost every cell of every multicellular organism, is evolutionarily well-conserved. Note that we have removed the letter “m” in the word “mTOR” to signify the fact that virtually the same pathway exists in non-mammal multicellular organisms. The results of mathematical analysis, being based on the backbone structure and being independent of the parameters, has value in its generality.

### 4.2. Physiologic and Pathologic Phenotypes

It is intuitive that the toggle switch (bistability), being in the middle region of Figure 3, corresponds to the normal functioning of the pathway. The middle region is flanked by the upper and lower regions, which may correspond to two kinds of pathology that are mechanistically opposite.

The upper region contains irreversible switches, where irreversibility implies that once Akt is activated, the activation persists, even after the complete withdrawal of the growth factor. Therefore, it may correspond to a cancer phenotype. Indeed, as introduced above, sustained activation of Akt propagates pro-survival and pro-growth signals, regardless of the growth factors, and is often associated with cancer incidence. The irreversible activation of Akt can be achieved by the increase of α (Figure 4). Because α = φ − ψ and φ is relatively constant, the increase of α is largely due to the decrease of ψ. Because ψ positively correlates with mTORC1 activity, mTORC1 inhibition would increase α and, thus, increase cancer risk. This provides valuable insight into the unpredictable results of mTORC1 inhibition in clinical trials for cancer treatment: while mTORC1 inhibition weakens cancer growth by several mechanisms (inhibiting macromolecular synthesis, inducing autophagy and diminishing the mTOR-dependent survival mechanism for chemotherapy [130]), it also perversely acts to promote cancer cell survival through constitutive activation of Akt.

The lower region contains response curves as gradually increasing functions, which usually do not dispose glucose rapidly enough after a meal, causing persistent hyperglycemia [1]. Therefore, responses in this region may correspond to insulin resistance and T2D phenotypes. This is also clear from the perspective of feedback loops. Under the assumption *K* → 0, the lower region can be characterized by α < 0 (the negative feedback dominates over the positive feedback), which makes Akt activation difficult, as well as the subsequent glucose uptake. The enhancement of negative feedback is often due to mTORC1 overactivity, which can be caused by gene mutations and nutritionally saturated states (high concentrations of amino acids, FFAs, TNFα, *etc.*).

Although normal responses should be within the middle region, this does not mean responses in the middle region are always normal. It is likely that normal responses only occupy a small area within the middle region (e.g., the green area in Figure 3). More importantly, the phase diagram is for *K* and α; thus, it cannot reflect the influence of the parameter β. On one hand, the decrease of β would shift the response curve to the right (Figure 5). This would raise the threshold of insulin response, and the resulting insulin resistance would increase the risk of T2D. Τhe decrease of β can be caused by factors such as FFAs and IL-6, which reduce the PI3K activity. On the other hand, Figure 5A tells us that when α > Θ_0_(*K*), the excess increase of β would cause the irreversible activation of Akt. Because the value of β positively correlates with E^P^/E^dP^, molecular events that increase E^P^ or decrease E^dP^ would increase β and, thus, increase cancer risk. The E^P^ increasing events include the loss of PTEN, gain-of-function *PIK3CA* mutation, over-activation of PI3K triggered by nicotine or viral agent PyMT. The E^dP^ decreasing events include PP2A inhibition by viral agents PyST or SVST and the overexpression of CIP2A. Finally, note that when α < Θ_0_(*K*), the increase of β would not lead to irreversible activation of Akt (Figure 5B).

Because the pathway deregulations are highly relevant to cancer and T2D, the phase diagram (*i.e.*, Figure 3) appears to be a credible description of what occurs *in vivo*, with the middle region corresponding to the normal phenotype. To further test this hypothesis, Wang considered the PI3K/Akt/mTOR pathway from a different perspective [129]. Instead of further studying the molecular level pathway dynamics, he shifted the focus to the organismal level by studying plasma glucose-insulin homeostasis, which is influenced both by the organismal level feedback mechanisms and by individual cells’ action (the pathway). By hypothesizing how the pathway should be designed in order to confer optimal glucose-insulin homeostasis, he proved that the toggle switch is the best mode of insulin action.

Although cancer and diabetes are opposite one another on the phase diagram, this does not mean that the two diseases are incompatible; it is more difficult for a cancer patient to contract T2D than a normal subject and *vice versa*. In fact, the opposing positions only imply that the two phenotypes are mechanistically opposite at the cellular level. For a multicellular organism like us, many cell types co-exist in the body, and there is no problem that the cancer phenotype exists in one cell type and the T2D phenotype exists in another. Moreover, the two diseases may be favorable to each other. For example, insulin resistance of muscle cells leads to hyperglycemia in a T2D patient. This high glucose concentration in the blood would be beneficial to the growth of cancer, which is known to be a glucose addict. Compared with normal subjects, T2D patients should have higher risks of cancer development. Further investigations on this possibility will be interesting.

Finally, although the upper and lower regions are assumed to be pathologic, they may correspond to normal regulatory mechanisms for certain species or cell types, under certain extreme conditions or during certain stages of development. Indeed, there are innumerable multicellular species, and different species live in disparate environments and have disparate physiologies. It is thus unlikely that a single toggle switch is responsible for the whole spectrum of metabolic conditions. That is, the monotone type and irreversible switch may be adopted by some species whose physiology is quite different from ours. Even for the same organism, the ever-changing metabolic conditions may also drive the deformation of the response curve, and it is possible that a qualitative transition occurs temporarily in order to cope with an extremely harsh condition. Furthermore, even for the same organism at a single time, it is possible that more than one kind of control mechanism coexists, due to a wide variety of cell types in the body.

### 4.3. Combination Therapy Guided by Computation

It was postulated that cancer, diabetes and health exist as stable states of the same dynamic system [131,132]. From this perspective, the essence of disease treatment is to induce the transition from the pathologic state to the healthy state. In terms of Figure 3, this corresponds to a transition of state points from a pathologic region to the green region, as exemplified by the dashed line in the figure. The abovementioned mathematical model can be employed to develop strategies to accomplish an efficient transition from one state to another [128]. Such strategies may include multiple-drug regimens targeting several specific points in the pathway, producing maximal synergistic effects, with minimized toxicity and drug resistance.

One insight obtained from mathematical analysis is the decoupling of β (whose influence is indicated by Figure 5) from *K* and α (whose influence is indicated by Figure 3). This decoupling specifically implies the necessity of combination therapy. The transition indicated by the dashed line in Figure 3, although rectifying aberrations in the *K* ~ α plane, would fail to rectify aberrations caused by an abnormal β. Conversely, the *K* ~ α plane aberrations would not be rectified by isolated adjustment of β. By fixing the value of *K*, the decoupling is essentially between α and β. It is now clear that α primarily determines the shape of the responding curve (Figure 4), whereas β primarily determines the position of the curve (Figure 5). In many cases, both α and β need to be targeted to adjust the response curve to normal. For example, FFAs both downregulate PI3K activity (reduce β) and upregulate mTORC1 (reduce α). Therefore, it is necessary to target both PI3K and mTORC1 to treat obesity-induced insulin resistance. Another example is cancer treatment. Many cancer phenotypes are characterized by both PI3K and mTORC1 hyperactivity. The mathematical analysis has revealed that the two molecules play different roles in carcinogenesis; and their targeting also has differential consequences. Therefore, targeting both is required, because they are irreplaceable of each other. The composition of such a drug regimen depends on future investigations, including the study of the detailed pharmacodynamics.

Another insight obtained from mathematical analysis is that [Akt]_total_, the total concentration of Akt, needs to be sufficiently large, so as to obtain a finely-tuned biologic response to so many inputs. This requirement is a consequence of the smallness requirement of *K*. Because *K = K_m_/*[Akt]_total_ and the Michaelis constant *K_m_* is relatively constant, [Akt]_total_ has to be sufficiently large to make *K* sufficiently small. This is an important message. Because Akt is a well-known oncoprotein, it seems natural to reduce [Akt]_total_ (by suppressing the mRNA expression of Akt) for cancer treatment. There are indeed many experimental and clinical practices under way in that direction [133]. The mathematical analysis, however, stresses that a large total Akt concentration is in fact a good thing; it is necessary for sensitive responses that are required by most biological functions. The inhibition of total Akt would enlarge *K*, which would, in turn, reduce sensitivity, degrade the quality of the pathway response and increase the risk of both cancer and T2D. To inhibit cancer growth, it is necessary to reduce pAkt, but one should not target the total Akt for that purpose. From a systematic perspective, it is predictable that direct inhibition of Akt expression may cause more severe toxicity than inhibition of its regulators, such as PI3K and mTOR [89,128].

Our discussion thus far may give the impression that α and β are mTORC1 and PI3K activities, respectively. In fact, α and β are composite parameters, which also involve molecules other than mTORC1 and PI3K. These molecules may also be targeted for combinatorial therapy.

### 4.4. The Yet to Be Explored “E^dP^” Wing

The enzyme group, E^dP^, has not been fully explored in the mathematical model. However, it can be predicted that the modulation of E^dP^ would render a parallel shift of the response curve, because the parameter β negatively correlates with E^dP^. That is, the increase (decrease) of E^dP^ would shift the response curve to the right (left). Although it is up to future data enrichment, the E^dP^ wing (as shown in Figure 1), which inactivates Akt by dephosphorylation, is subject to far less feedback control than the E^P^ wing. If well harnessed, there may be strong therapeutic value in the E^dP^ wing. The E^dP^ wing contains PP2A and PHLPP; PP2A is better studied, because of its expected role as a tumor suppressor [12,20,55,134].

To date, most of the efforts aimed at correcting the over-activation of Akt-related signaling has been focused on the kinases that promote Akt activation, such as PI3K, mTORC2 and IRS1. Comparatively, less attention has been invested in the phosphatases, which deactivate Akt. Admittedly, the sheer number of phosphatases makes study and intervention a formidable challenge. For example, PP2A, a major Akt phosphatase, is in reality a set of over 90 different PP2A holoenzymes, each likely to have a number of different substrates [135]. Therefore, one must be careful as to which specific type of PP2A holoenzyme to target and which substrates are affected. In spite of this complexity, inhibition of PP2A activity, either by exogenous factors, like toxicants and viral agents, or by cellular regulation through SET, CIP2A and α4 protein, is frequently associated with tumorigenesis [134,136,137,138]. PP2A has thus been recognized as a tumor suppressor, and the restoration of normal PP2A activity may be therapeutically useful [12,139]. PP2A could place essential control on the proliferation and survival of malignant cells, not only by inhibiting Akt, but also by inhibiting the oncoprotein, c-Myc. These two beneficial PP2A effects are suppressed in malignant cells, where CIP2A tends to accumulate and directly inhibit the anti-tumor effects of PP2A [140]. Therefore, the restoration of PP2A function in malignant cells could be an effective approach for cancer therapy.

One prominent option for PP2A enhancement in malignant cells would be to quench the expression of CIP2A. The robust expression of CIP2A is a causative factor of many types of human cancer and is associated with resistance to chemotherapy [57,134]. In a mouse model evaluating the absence of CIP2A, the CIP2A mutant mice showed no defects in growth or development, suggesting CIP2A targeting could be associated with less morbidity than direct action on the PAM pathway [140]. Progress has been made by using small interference RNA (siRNA) to knock down CIP2A expression in breast cancer cells and NSCLC (non-small cell lung cancer) cells, both decreasing the tumorigenic potentials of the cancer cells and sensitizing them to chemotherapy [136,141]. However, challenges still remain to identify which specific types of PP2A and their substrates are affected by targeting CIP2A. Further, the discovery of other CIP2A inhibitors, as well as enrichment of *in vivo* data will go a long way in guiding this therapeutic strategy [140].

### 4.5. Patient Selection Guided by Computation

A deliberation of patient selection can help some to avoid serious adverse effects [7]. For example, a new benchmark to analyze the potentially beneficial and toxic effects can sift out patients who are not suitable for certain therapy. Thus far, gene mutation analysis is available to help predict the would-be sensitivity of a patient to certain targeted therapy [142]. Furthermore, diabetes patients and pre-diabetic subjects may be at greater risk for metabolic toxicity in PI3K/Akt/mTOR targeted therapy, although the molecular basis for such discrimination is unclear [108].

The phase diagram (Figure 3) can provide a global picture of the responses of body cells of a patient or a normal subject. Certain types of cell can be extracted from a patient, such as muscles, fat, cells in the blood and cells obtained from biopsy. One can then examine many of these cells by determining their response curves and then locate them on the phase diagram. In this way, a map of the subject’s cellular response can be obtained; and the pattern of the map can be analyzed. From this map, one can evaluate the health status of the subject in terms of the PI3K/Akt/mTOR pathway. For example, a pre-diabetic subject may appear to be healthy, but the map would reveal that his/her muscle cells are insulin resistant. One can also predict the global consequences of certain treatments for a patient. If a cancer patient is fortuitously found (by the map) to be pre-diabetic, then certain cancer treatments (e.g., inhibiting PI3K) could be dangerous, because the treatments could induce severe metabolic toxicity, such as hyperglycemia. The global analysis of the map would suggest a more detailed scheme that could render a more targeted treatment, while at the same time minimizing global side effects as much as possible.

## 5. Molecular Dynamics Simulation Inspires a New Generation of Therapeutic Agents

While systems-level modeling of the PI3K/Akt/mTOR pathway provides insights into the strategy of combining inhibitors for effective therapy with reduced toxicity, molecular-level modeling may help discover inhibitors with higher specificity and efficacy. Indeed, the computer-aided study of targeting sites of the kinases is a powerful tool for drug discovery.

It is widely established that based on nuclear magnetic resonance (NMR) and X-ray crystallographic structures, well-defined binding pockets can be revealed by molecular modeling [143]. Yet, unlike traditional crystallographic studies that demonstrate protein flexibility based on extensive labor and capital expense, molecular dynamics (MD) simulations use principles of classical/quantum mechanics to calculate atomic forces, which govern the molecular movement, in order to simulate protein flexibility and dynamics [144]. MD simulation is thus useful for studying the conformational space accessible to proteins and protein-ligand complexes, uncover drug-targetable allosteric sites and reveal cryptic binding sites that are usually overlooked by traditional crystallographic studies [145]. MD simulation can also be employed to examine the strength of docked protein-ligand conformations, reveal transient binding sites and explore altered drug binding profiles of protein variants [146]. Further development of MD simulations offer various techniques, such as thermodynamic integration, single-step perturbation and free energy perturbation, all serving as powerful tools in drug discovery and optimization [146]. In the following, we take PI3K as an example to show the use of computer-aided analysis in isoform-specific and mutation-specific inhibitor discovery.

In total, there are eight PI3K enzymes, which are grouped into three classes, among which, class I is the most important to cancer. Class I PI3K consists of four members, PI3Kα, PI3Kβ, PI3Kγ and PI3Kδ. They are each comprised of a 110-kDa catalytic subunit, p110α, p110β, p110γ and p110δ and a regulatory subunit. The activation mechanisms of the four isoforms of PI3K are different, yet they crosstalk with one another [104]. The first generation of PI3K inhibitors developed were pan-class I PI3K inhibitors, which inhibited all four members of class I PI3K activity [10]. Later studies illustrated different structures of the four catalytic subunits. Thereby, computational visualization and analysis of the different structures offered insights into isoform-specific PI3K inhibition [143]. Nonetheless, one study argues that the use of pan-PI3K inhibitors is necessary, because any class I PI3K activation can sustain cell survival and proliferation, due to the redundancy of the PI3K isoforms [147]. However, the pan-PI3K inhibitors exert relatively severe toxicity, both on-target and off-target, compared to isoform-selective inhibitors. Thus, the dose limitations of pan-PI3K inhibitors prohibit them from achieving desired therapeutic effects [104]. It is prudent to target the malfunctioning isoforms of PI3K, without perturbation of the other normal isoforms. Indeed, evidences from experimental and clinical studies show that different isoforms of PI3K are activated in different circumstances and have differential functions. For example, PI3Kα is the isoform with high rates of mutation, whereas PI3Kβ is the isoform that is overactivated at *PTEN* mutation [148,149]; PI3Kγ is important for stress-induced pathological, but not physiological, cardiac hypertrophy [17]; PI3Kδ is highly expressed in lymphocytes and highly involved in B- and T-cell responses [150]. In addition, isoform-specific PI3K inhibitors have the potential to completely inhibit the activity of the target with tolerable toxicity [104].

MD simulation and its subsequent studies provided valuable tools for the development of isoform-specific PI3K inhibitors, complementing the molecular modeling method [151]. It has been reported that the combinatory effort of comparative molecular field analysis (CoMFA), comparative molecular similarity induces analysis (CoMSIA), docking analysis and MD simulation can elucidate the structure-activity correlation of the inhibitors of p110α, as well as the binding modes of inhibitors at the ATP binding pocket [152]. Furthermore, the free energy calculations are used to select and confirm the isoform-specific inhibitors to p110α, p110β or p110δ, *in silico* [153,154]. So far, ten PI3K inhibitors of high isoform specificity are under clinical trials [104]. For example, the p110α-specific inhibitors, NVP-BYL719 and MLN1117 (previously known as INK1117), show selective inhibitory potency to p110α, 50- and 100-fold higher than other class I PI3K isoforms, respectively [155,156]; p110β-specific inhibitor SAR260301 has shown 88- and 26-fold selectivity, respectively, over p110α and p110δ [148]; GS-1101, a p110δ-specific inhibitor, shows 40- to 300-fold selectivity against other isoforms [157]. Further studies of the isoform-specific inhibitors prove that GS-1101 effectively inhibits the activity of PI3Kδ with tolerable doses and can produce a drastic response in B-cell malignancies [158], whereas NVP-BYL719 and MLN1117, in combination with EGFR and mTOR inhibitors, respectively, have also shown promising antitumor efficacy in recent studies [159,160].

Of the four class I PI3Ks, only PI3Kα has been frequently mutated in human cancers [149]. Three hotspots for p110α mutations, H1047R, E545K and E542K, all increase PI3K activity [104]. Therefore, a study of the conformation changes based on the mutations could lead to the design of suitable drugs specifically targeting mutated PI3K. Encouragingly, an *in silico* docking and MD simulation study of 33 PI3K inhibitors binding to conformations of the wild-type and mutant PI3Kα has demonstrated the conformation differences between an H1047 mutation and wild-type p110α for mutant-PI3Kα-specific drug design [161]. Other studies using structure-based drug design led to the development of mutated-PI3Kα targeted inhibitors, such as CH5132799 and DW09849 [162,163]. The drug efficacy of such new-generation inhibitors is in the process of characterization. For example, CH5132799 shows potency to sensitize *PIK3CA*-mutated xenograft tumor to chemotherapy [164]. Further studies of the toxicity profile are also expected.

There are also studies using MD simulation to explore inhibitors for other components in the PI3K/Akt/mTOR pathway, such as Akt and mTOR [165,166,167,168]. However, in contrast to PI3K, MD studies of the other components in the pathway are sparse and less extensive. MD simulation is a rapidly developing field and is likely to play an increasingly important role in drug discovery [144,145]. Therefore, it is important to perform MD simulation on other components of the pathway, including mTOR, IRS and the interaction between IRS and PI3K.

## 6. Conclusions

The PI3K/Akt/mTOR pathway has essential pro-survival and pro-growth functions in the cell, amongst a variety of other cellular functions. The perturbation of this important pathway is associated with many diseases. We reviewed a number of alterations of this pathway and the pathological consequences, including genetic mutations, viruses, chemical toxicants, metabolites and inflammatory factors. Over-activation of this pathway is essential for the survival and proliferation of the malignant cells and is therefore a feature of many cancers. Toxicology studies find that the PI3K/Akt/mTOR pathway is commonly targeted by toxins and viruses to produce disease, but also suggest that the pathway is pharmaceutically modifiable. A new generation of therapy, aiming at rectifying the pathological alterations of this pathway, is under vigorous development. It has already been demonstrated that PI3K/Akt/mTOR targeted therapy inhibits components of the pathway and shows therapeutic efficacy, albeit with a variety of toxic side effects. Thus, a thorough understanding of this pathway, with a systemic perspective and computational modeling, is important. Here, we reviewed the current status of computational-aided targeting of this pathway, including mathematical modeling at the system level, as well as MD simulations at the molecular level. For future applications, further analysis of the dynamics of normal and pathologically-altered pathway signaling, coupled with pharmacodynamics characterizations, presents new opportunities to compose therapy optimized for rectification of the aberrant pathway. Computational analysis of patient cell sampling may help predict metabolic toxicity by molecular targeted inhibition. Finally, *in silico* simulation of different isoforms and mutated conformations of the pathway components may help design more highly selective drugs, achieving better therapeutic effects with less toxicity.
